# Chia (*Salvia hispanica* L.) Flour and Oil Ameliorate Metabolic Disorders in the Liver of Rats Fed a High-Fat and High Fructose Diet

**DOI:** 10.3390/foods11030285

**Published:** 2022-01-21

**Authors:** Luiza de Paula Dias Moreira, Bárbara Nery Enes, Vinícius Parzanini Brilhante de São José, Renata Celi Lopes Toledo, Luiz Carlos Maia Ladeira, Rodrigo Rezende Cardoso, Vinícius da Silva Duarte, Helen Hermana Miranda Hermsdorff, Frederico Augusto Ribeiro de Barros, Hércia Stampini Duarte Martino

**Affiliations:** 1 Department of Nutrition and Health, Universidade Federal de Viçosa, Viçosa 36570-900, Brazil; luizamdp20@gmail.com (L.d.P.D.M.); barbara.nery.enes@gmail.com (B.N.E.); v_brilhante@hotmail.com (V.P.B.d.S.J.); renatacelly@yahoo.com.br (R.C.L.T.); helenhermana@ufv.br (H.H.M.H.); 2 Department of General Biology, Universidade Federal de Viçosa, Viçosa 36570-900, Brazil; luizmaialadeira@gmail.com; 3 Department of Food Technology, Universidade Federal de Viçosa, Viçosa 36570-900, Brazil; rodrigo17cd@yahoo.com.br (R.R.C.); fredbarros@ufv.br (F.A.R.d.B.); 4 Faculty of Chemistry, Biotechnology, and Food Science, The Norwegian University of Live Sciences, 1432 Ås, Norway; vinicius.da.silva.duarte@nmbu.no

**Keywords:** oxidative stress, lipogenesis, liver steatosis, inflammation

## Abstract

We hypothesized that the consumption of chia (*Salvia hispanica* L.) flour (CF) and chia oil (CO) improves metabolic disorders in the liver of Wistar rats (*Rattus norvegicus domestica*) fed a high-fat and high-fructose (HFHF) diet. The animals were fed a HFHF diet (*n* = 30) or AIN93-M standard diet (*n* = 10) for eight weeks. After this period, the animals fed HFHF were divided into three groups (*n* = 10): HFHF diet, HFHF plus 14.7% of CF, and HFHF plus 4% of CO. Histological and biochemical analyses, gene expression, protein levels related to inflammation, and oxidative stress were evaluated in the liver. The HFHF diet caused lipogenesis, liver steatosis, oxidative stress, and inflammation in the animals. The CF and CO intake increased the liver total antioxidant capacity and superoxide dismutase, decreased nitric oxide levels and liver steatosis. Furthermore, the CF and CO led to the upregulation of *Cpt1a* and *Adipor2*, respectively, whereas CF downregulated *Srebf1*. CO intake decreased blood glucose, triglycerides, and the animals’ body weight. Chia did not show effects on mitigating liver pro-inflammatory status, which it may indicate occurs later. The addition of chia into an unbalanced diet is a good and relevant strategy to reduce liver metabolic disorders caused by the high consumption of fructose and saturated fat.

## 1. Introduction

High consumption of fructose and saturated fat is related to an increase of non-communicable chronic diseases, such as obesity, insulin resistance, metabolic syndrome, type 2 diabetes, and mainly, nonalcoholic fatty liver disease (NAFLD). Several organs are affected, although it has been mostly related to metabolic dysregulation and liver-centered conditions. Liver injury can advance to a specific histological phenotype such as hepatocyte ballooning, macrovesicular steatosis, lobular inflammation, fibrosis, cirrhosis, and hepatocellular carcinoma, which makes it a worldwide public health problem [1,2,3].

Liver dysregulations may cause insulin resistance, oxidative stress, inflammation, and enhance lipogenesis and beta-oxidation. The synthesis of fatty acids by the excess of glucose and mainly fructose is called *de novo* lipogenesis, which leads to depletion of adenosine triphosphate in the liver. It contributes to cellular stress and increases the risk of developing liver-related complications [4]. Primarily, high intakes of carbohydrates and fatty acids cause saturation of the oxidative pathway. In addition, the increase of adipose tissue is regulated by insulin, which leads to lipolysis of triglycerides (TG), increasing free fatty acids influx, synthesis and retention of lipid in hepatocytes, and the formation of lipotoxic compounds. Besides, these mechanisms can alter the enzymatic antioxidant system and increase lipid peroxidation causing a redox imbalance.

Lipogenesis rates are mainly up-regulated by sterol regulatory element binding transcription factor 1 (SREBP1c)) (synonym *Srebf1*) and acetyl-CoA carboxylase 1 (*Acc1*) associated with down-regulation of genes involving fatty acid oxidation like carnitine palmitoyltransferase 1a (*Cpt1a*), adiponectin receptor 2 (*Adipor2*), and proliferator-activated receptor alpha (PPAR-α) [1,5,6]. Moreover, a systemic and tissue proinflammatory state is also observed in the NAFLD by the activation of the Toll-like Receptor 4 (TLR4)/Nuclear Factor Kappa B (NF-κB) signaling pathways and increased levels of pro-inflammatory cytokines [7]. These metabolic disorders contribute to hyperglycemia and consequent insulin resistance. Furthermore, high levels of hepatic enzymes, such as alanine aminotransferase (ALT) and aspartate aminotransferase (AST), and high levels of uric acid in the liver can indicate liver damage [8,9].

In this sense, the consumption of functional foods and/or their isolated bioactive compounds can reduce the risk of developing metabolic liver disorders and assist in the treatment of NAFLD (Li et al., 2021). From this perspective, the chia seed (*Salvia hispanica* L.) stands out for its high functional potential exemplified by its dietary fiber, lipids, mainly α-linolenic fatty acid (ALA), proteins, vitamins, minerals, and bioactive compounds [10,11]. Studies conducted in in vitro models, with animals, and a few clinical trials have demonstrated the benefits of chia [12,13,14]. A systematic review carried out by our research group [15] demonstrated that the inclusion of chia (whole seed, flour, or oil) into unbalanced diets can reduce several metabolic disorders. Chia oil (4% *v*/*v*) was also able to reduce body adiposity and insulin resistance in rats fed an HFHF diet [16]. However, the effects of chia on metabolic liver disorders, mainly lipogenesis, oxidative stress, and inflammation in rats fed a HFHF diet remain unclear. Therefore, we hypothesized that the consumption of chia (*Salvia hispanica* L.) flour (CF) and chia oil (CO) improve lipogenesis, fatty acid oxidation, liver steatosis, oxidative stress, and inflammation in the liver of rats fed a high-fat and high-fructose (HFHF) diet. The aim of this study was to evaluate the effects of CF and CO on oxidative stress, liver steatosis, gene expression related to lipogenesis and fatty acid oxidation, and inflammation in the liver of rats fed HFHF.

## 2. Materials and Methods

### 2.1. Chia Characterization

#### 2.1.1. Raw Material

The chia seeds used in this study were cultivated in the state of Rio Grande do Sul, Brazil. The seeds were stored at −20 °C until further analysis. The seeds were milled every 15 days in a blender (Mondial^®^ -model NL-26, Conceição do Jacuípe, (Bahia), Brazil) at level 2, for 3 min, and stored in vacuum packaging at −20 °C.

#### 2.1.2. Chia Oil Extraction

The chia oil was obtained weekly through the cold pressing of chia flour by using a hydraulic mechanical press (Carver Laboratory Press, Model C 22400-36, Summit, NJ, USA). The oil was collected, filtered, centrifuged at 1050× *g*, for 15 min at 7 °C, and stored in amber glass at −20 °C.

#### 2.1.3. Chia Proximate Composition

Chia proximate composition (moisture, ash, proteins, total lipids, dietary fiber, and carbohydrates) was determined by the methods recommended by the Association of Official Agricultural Chemists (AOAC) [17].

#### 2.1.4. Determination of Total Antioxidant Capacity and Total Phenolic of Chia Flour

The extracts were prepared by adding 2 g of chia flour into 20 mL of acetone solution (70% acetone/water). The supernatant was shaken (2 h, 25 °C), centrifuged (2865× *g*, 15 min (Hermle^®^, model Z216MK, Wehingen, Germany), transferred to a beaker and with volume made up to 20 mL with acetone (70% acetone/water). Determination of the free radical scavenging capacity of 2,2-diphenyl-1-picrilhydrazil (DPPH) was performed and the anti-radical activity was expressed in µmol equivalent to Trolox/g of the sample (µmol Trolox/g) [18]. The total phenolic content of the chia flour was determined according to the Folin–Ciocalteu method [19]. The absorbance was read at 765 nm on a spectrophotometer (Thermo Scientific, Evolution 606, Madison, WI, USA). A standard curve ranging from 0 to 300 ppm of gallic acid (GA), and the results were expressed in mg of gallic acid equivalents/mL of extract of chia flour (mg GAE/mL).

#### 2.1.5. Peroxide and Acidity Content, and Fatty Acid Profile of Chia Oil

The chia oil peroxide value was performed according to the procedures recommended by American Oil Chemists Society [20]. The result was expressed as milliequivalents (mEq) of peroxide/1000 g of oil sample. Acidity was determined and the results were expressed per mg of KOH/g of oil [21]. The fatty acids composition of fatty acids of chia oil was determined by gas chromatography, adapted for vegetable oil [22]. Then, 100 µL of the chia oil was added to 4 mL of 0.5 M KOH in methanol for basic catalysis for 30 min, vortexed each 5 min. After the reaction, 2 mL of distilled water was added. Following this, 5 mL of hexane (Honeywell—Riedel-de Haen) was added for the extraction of fatty acid methyl esters. The samples were centrifuged (Quimis Centrifuge) at 1050× *g* for 2 min, and the organic phase was collected. After, 0.3 g of anhydrous sodium sulfate was added to the organic phase to absorb the remaining water. The material was filtered (Teflon membrane (PTFE), 22 μm) and poured into a vial tube. The methyl esters were analyzed in a gas chromatograph (Shimadzu GC-2010) with an automatic injector, using a flame ionization detection system (FID), using a capillary column of fused silica SP-2560, with an 0.18 mm internal column diameter and the 75 m length. The identification of fatty acids was compared through the retention time of fatty acids in the samples F.A.M.E. Mix C14-C22-Sigma-Aldric, St. Louis, MO, USA), using hexane as a solvent.

### 2.2. Animals and Experimental Design

#### 2.2.1. Animals

The study was carried out with forty male Wistar rats (*Rattus norvegicus domestica*), 45–50 days old. The study protocol was approved by the Ethics Commission on Animal Use (CEUA) at the Federal University of Viçosa (CEUA/UFV—protocol no. 89/2018; date of approval: 21 February 2019) and followed the university guidelines for animal use for Animal Biomedical Research. The rats were obtained from the Central Animal Facility of the Center for Biological Sciences and Health at the Federal University of Viçosa, Minas Gerais, Brazil. The animals were kept in individual stainless-steel cages, under controlled conditions (22 °C ± 2 °C, 12:12-h light–dark cycle).

#### 2.2.2. Experimental Design and Diets

**Figure 1:**
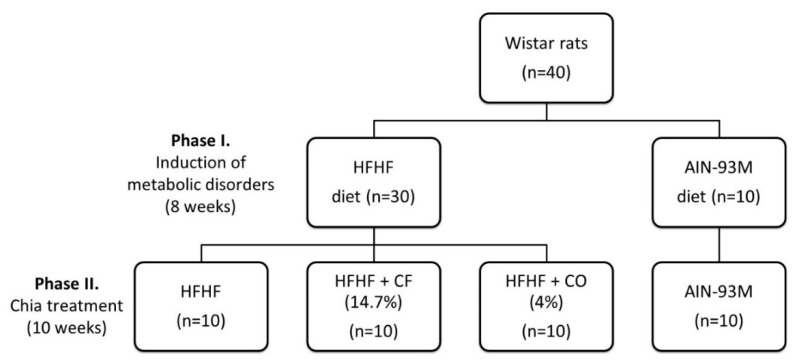
Experimental design. Phase I: Animals were fed a high-fat and high-fructose (HFHF) diet (*n* = 30) containing 4% (*w*/*w*) soybean oil, 31% (*w*/*w*) lard and 20% fructose (*w*/*w*), or an AIN-93M diet (*n* = 10) for eight weeks. In the chia treatments (phase II), the HFHF group was divided into three groups (*n* = 10): HFHF diet: the animals kept the HFHF diet; chia flour (CF) treatment group: HFHF diet with 4% of soybean oil replaced by chia flour lipid (14.7% of chia flour); and chia oil (CO) treatment group: HFHF diet with 4% of soybean oil replaced by 4% of chia oil for ten weeks; and the AIN-93M group: the animals kept the AIN-93M diet for ten weeks.

**Figure 2:**
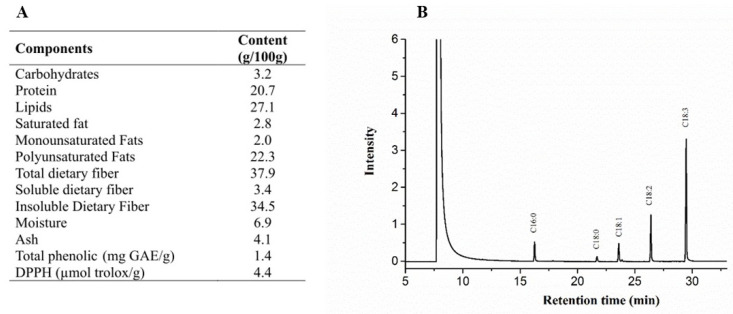
(**A**) Chemical characterization of chia flour. (**B**) Chia oil chromatogram with identification of fatty acids: P1: palmitic acid (C16:0, 7.6%); P2: stearic acid (C18:0, 2.7%); P3: oleic acid (C18:1, 7.5%); P4: linoleic acid (C18:2, 19.9%); and P5: alpha linolenic acid (C18:3, 62.3%). In total, 10.3% of fatty acids are saturated, 7.5% monounsaturated, and 82.2% polyunsaturated.

The experimental period was divided into two phases (Figure 1). In the first one (eight weeks), the rats were randomized by body weight (b.w.) into two groups and received a modified standard diet AIN93-M (AIN93-M group) [16], b.w. = 156.0 ± 17.0 g, *n* = 10; or high-fat and high-fructose diet (HFHF group; b.w. = 156.5 ± 17.9 g, *n* = 30) for eight weeks. The HFHF diet contained 4% (*w*/*w*) of soybean oil, 31% (*w*/*w*) of lard, and 20% (*w*/*w*) of fructose [16]. After this period, the animals fed the HFHF diet were randomized according to body weight into three different groups (*n* = 10): control: HFHF (b.w. = 366.8 ± 35 g); chia oil (CO): HFHF diet plus chia oil (CO) (b.w. = 362.5 ± 34.8 g); or chia flour (CF) (b.w. = 362.4 ± 35.4 g): HFHF plus CF 14.7% of chia flour, for ten weeks. The vitamins and minerals mix of the diets were administrated equally for all diets, as well as the quantity of macronutrients, following the recommendation of a standard diet AIN93-M, with modifications: the replacement of protein source casein for albumin. To calculate the chia treatment diets, soybean oil (4%) was replaced by chia oil (4%) (CO diet); or 4% lipid from chia flour, which is equivalent to 14.7% of chia flour (CF diet). To calculate the CF diet, chia flour chemical composition was considered (Figure 2A). Since 14.7% of chia flour contains 5.58% of dietary fiber, the other diets (AIN93-M, HFHF, and CO) contained the same amount of microcristalline cellulose (Table 1). The caloric density was determined by the conversion factors of 4 kcal/g for carbohydrates and proteins and 9 kcal/g for total lipids. The diets were prepared every 15 days, packed in polyethylene bags, and stored at −20 °C. The group-specific diets and distilled water were offered *ad libitum* during the experimental period.

#### 2.2.3. Food Intake, Biometric Parameters, and Euthanasia

The food intake and body weight were measured weekly. At the end of phase II, after ten weeks of the experiment, the naso-anal length (NAL) was measured by the Lee index, calculated using the equation: $\sqrt[{3}]{b.\;w.\;\left(g\right)}$/NAL (cm) × 1000.

At the end of the experiment, the animals were exposed to isoflurane general inhalation anesthesia (Isoforine, Cristália^®^, São Paulo, Brazil), and then the euthanasia was performed by exsanguination through cardiac puncture. The blood was immediately collected by cardiac puncture (no fasting period), and centrifuged at 1006× *g* for 10 min, 4 °C (Hettich Universal 320 R, São Paulo, Brazil) for the biochemical analysis. The liver was rapidly collected, weighed, and a liver fragment was dissected and immersed in 10% formaldehyde. The remaining organ was immediately frozen in liquid nitrogen, and then stored at −80 °C for further analyses. The hepatosomatic index was calculated using the following equation: liver weight (g)/b. w. (g) × 100.

### 2.3. Biochemical Analysis

The blood glucose and TG concentrations were measured by handheld monitors Accu-Chek^®^, and Accutrend^®^ GCT, respectively (Roche, Diabetes Care Ltd., São Paulo, Brazil), by the caudal vein puncture after ten weeks. The plasma was used to measure the concentrations of aspartate aminotransferase (AST), alanine aminotransferase (ALT), and uric acid using commercially available kits (Bioclin^®^, Belo Horizonte, Brazil) by BS-200 Chemistry Analyzer, Bioclin^®^.

### 2.4. Gene Expression in the Liver

The mRNA expression levels of genes related to lipogenesis: *Srebf1* (sterol regulatory element binding transcription factor 1), and fatty acid oxidation: *Cpt1a* (carnitine palmitoyltransferase I) and *Adipor2* (adiponectin receptor 2) were evaluated in the liver by quantitative reverse transcriptase-polymerase chain reaction (RT-qPCR). Total RNA extraction was performed using Trizol Reagent (Invitrogen, CA, USA). The quantification of extracted mRNA was performed by a spectrophotometer (Multiskan™ GO spectrophotometer, Thermo Fisher Scientific; Waltham, MA, USA). An M-MLV Reverse Transcriptase Kit (Invitrogen, CA, USA) was used for cDNA synthesis. The gene expression relative quantification was performed by AB StepOne Real-Time PCR System equipment and Fast SYBR Green Master Mix (Applied Biosystems, Waltham, MA, USA) reagent. The initial parameters used were 20 s at 95 °C, then 40 cycles of 3 s at 95 °C, and lastly 30 s at 60 °C for annealing and extension. The primers used for amplification are listed as follows: *Srebf1*: CGC TAC CGT TCC TCT ATC AAT GAC (Forward); AGT TTC TGG TTG CTG TGC TGT AAG (Reverse); *Cpt1a*: GTA AGG CCA CTG ATG AAG GAA GA (Forward); ATT TGG GTC CGA GGT TGA CA (Reverse); *Adipor2*: CAT GTT TGC CAC CCC TCA GTA (Forward); ATG CAA GGT AGG GAT GAT TCC A (Reverse), and the housekeeping gene GAPDH (glyceraldehyde-3-phosphate dehydrogenase): AGG TTG TCT CCT GTC ACT TC (Forward); CTG TTG CTG TAG CCA TAT TC (Reverse). The primers (Sigma-Aldrich, São Paulo, Brazil) were designed by using the Primer 3 Plus program.Gene expression was calculated using the 2-Delta-Delta C (T) (2^−ΔΔCt^) method [23], by using GAPDH as endogenous control, and the HFHF group as the control normalized to 1.

### 2.5. Determination of Pro and Anti-Inflammatory Liver Proteins

Pro-inflammatory proteins: TNF (88-7340-88), (Fisher Scientific, Vienna, Austria); TLR4 (E-EL-R0990); PPAR-α (E-EL-R0725); *p65*-NF-κB (E-EL-R0674); and anti-inflammatory (IL-10: E-EL-R0016) (Elabscience, Houston, TX, USA) were determined by enzyme-linked immunosorbent assay (ELISA) kits for rats. To quantify the above-mentioned proteins, the liver homogenate was prepared according to NE-PER Nuclear and Cytoplasmic Extraction Reagents Kit (code 78835; Thermo Scientific, Vienna, Austria), except for IL-10, where 200 mg of liver tissue was macerated with 50 mM phosphate buffer containing 1 mM EDTA (pH 7.4) and centrifugated at 12,000× *g*, 4 °C for 10 min. The ELISA 96-well plates from the ELISA kits were precoated according to specific antibodies to each specific kit. The optical density was performed using the Multiskan™ GO spectrophotometer (Thermo Fisher Scientific; Waltham, MA, USA), with wavelength (nm),and results expressed according to each kit. The data were normalized to total liver protein calculated by the Bradford method [24].

### 2.6. Evaluation of Oxidative Stress

The total antioxidant capacity (TAC) of the liver and plasma, the activity of antioxidant enzyme superoxide dismutase (SOD), and the nitric oxide (NO) lipid peroxidation biomarker were evaluated in the liver. The total liver protein [24] was used to express the results of SOD concentrations. To obtain liver homogenate, 200 mg of liver tissue was macerated with 50 mM phosphate buffer containing 1 mM EDTA (pH 7.4), and centrifugated at 12,000× *g*, 4 °C for 10 min. The supernatant was recovered and stored at −80 °C.

#### 2.6.1. Total Antioxidant Capacity

TAC was evaluated in the liver homogenate and plasma samples following a commercial antioxidant assay kit (Sigma-Aldrich, St. Louis, MO, USA). A volume of 10 µL of each sample was transferred to a 96-well plate, followed by the addition of 20 µL of a myoglobin-containing solution, and 150 µL of 2,2′-azino-bis (3-ethylbenzothiazoline-6-sulfonic acid substrate (ABTS). The plate was incubated for 5 min at room temperature. Then, 100 µL of stop solution was added to each well, and absorbance was measured at 450 nm (Multiskan™ GO spectrophotometer, Thermo Fisher Scientific; Waltham, MA, USA). The results were expressed as nM Trolox equivalents.

#### 2.6.2. Superoxide Dismutase

SOD activity was quantified in relative units as the amount of enzyme required to inhibit 50% of pyrogallol oxidation under test conditions [25]. The absorbance was measured at 570 nm (Multiskan™ GO spectrophotometer, Thermo Fisher Scientific; Waltham, MA, USA), and the results were expressed as units of SOD activity/mg of liver protein.

#### 2.6.3. Nitric Oxide

Fifty microliters of liver homogenate was mixed with solutions A (1% sulfanilamide in 2.5% orthophosphoric acid (H_3_PO_4_) and B (0.1% naphthyl l ethylene diamide dihydrochloride in 2.5% H_3_PO_4_) (1:1), incubated in the dark for 10 min. The microtiter plate was read at 570 nm (Multiskan™ GO spectrophotometer, Thermo Fisher Scientific; Waltham, MA, USA) [26]. The NO concentration was calculated according to a standard curve ranging from 100 to 0 µM and the results were expressed in µmol.

### 2.7. Liver Histomorphometry

Liver tissue fragments were dissected and fixed in 10% formaldehyde for 48 h. The material was dehydrated using ethanol and embedded in historesin (Leica^®^, Wetzlar, Germany). A semi-automatic rotary microtome (Leica ^®^M2255) and glass knives (Leica^®^) were used for semi-serial cuts 3 μm thick, respecting the distance of 12 cuts between them. The images of the sections were taken under a bright field microscope (Olympus AX 70 TRF, Tokyo, Japan) with 20X objective. The inflammatory infiltrates and lipid vesicles were counted in the photographs from the sections stained with Gomori Trichrome using the Image J^®^ 1.48v (National Institutes of Health, Bethesda, MD, USA) software. In each histological field, a 266-point reticle was used until the sum of 1064 points per animal (*n* = 8) [27]. Picrosirius red staining was used to measure type I, III, and total collagen with a polarizing filter. The degree of liver steatosis was assessed quantitatively according to the 5-degree scale: Grade 0, if the percentage of fat was absent or <5%; Grade 1, if ≥5% and <25%; Grade 2, if ≥25% and <50%; Grade 3, if ≥50% and <75%; and Grade 4, if ≥75% [28]. The NAFLD activity score (NAS) was calculated based on the individual steatosis scores (0–3 points), lobular inflammation (0–3 points), and hepatocyte ballooning (0–2 points) in a blinded manner to assess the severity of NAFLD. A score greater than or equal to five was considered NASH [29].

### 2.8. Statistical Analyses

The Shapiro–Wilk test was used to evaluate normality data. Data were expressed as average ± standard deviation. The parametric data were evaluated by the one-way analysis of variance (ANOVA) and Newman-Keuls and Tukey (gene expression) as *post-hoc* tests to compare differences among groups. Kruskal-Wallis followed by Dunn’s *post-hoc* test was used for the non-parametric data. A significance level of 5% was considered. The statistical analyses and graphs were performed using GraphPad Prism software version 7.0 (San Diego, CA, USA).

## 3. Results

### 3.1. Chemical Composition of Chia

The chia chemical composition is depicted in Figure 2A. Among chia’s oil lipids, 10.3% were saturated, 7.5% monounsaturated, and 82.2% polyunsaturated. Regarding the fatty acid profile, 62.3% were alpha-linolenic acid (ALA) (C18:3), 19.9% linoleic acid (C18:2), 7.6% palmitic acid (C16:0), 7.5% oleic acid (C18: 1), and 2.7% stearic acid (C18:0) (Figure 2B). The peroxide value of chia oil was 1.9 mqE, and its acidity was 3.8 mg KOH/g.

### 3.2. Effect of Chia on Food Intake, and Murinometric Measures

**Figure 3:**
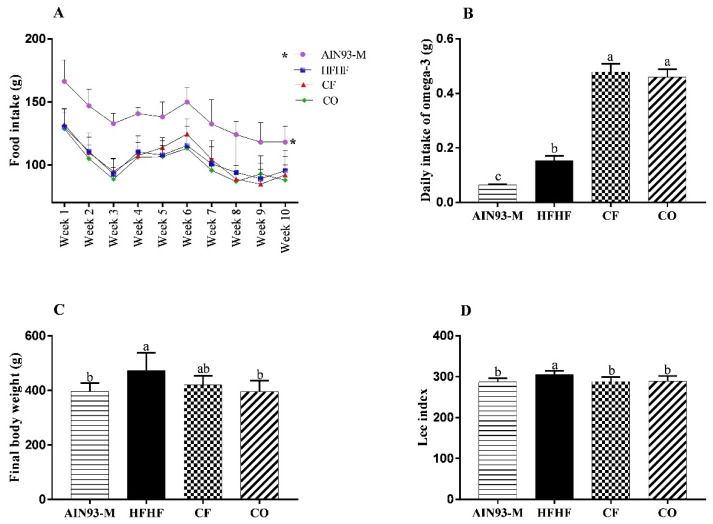
(**A**) Food intake (weekly); (**B**) Daily intake of omega-3; (**C**) Final body weight at the end of 10 weeks; (**D**) Lee index. The animals were fed the following diets: AIN93-M: standard rodent diet; HFHF: high-fat and high-fructose; chia flour (CF): HFHF with 4% of soybean oil replaced by chia flour lipid (14.7% of chia flour); and chia oil (CO): HFHF diet with 4% of soybean oil replaced by 4% of chia oil for 10 weeks. Values are represented by average and standard deviation (*n* = 10). The difference between the averages was analyzed by the one-way ANOVA test followed by the Newman–Keuls *post-hoc* test, except for daily intake of omega-3 (non-parametric data) performed by the Kruskal–Wallis test and the *post-hoc* Dunn’s test. * Food intake (**A**) indicates statistical difference between AIN93-M and the other groups. Different letters indicate significant statistic difference (*p* < 0.05).

The addition of CO and CF into the HFHF diet decreased the omega6:omega3 ratio from 5.77:1 to 1.46:1, while the AIN93-M diet showed a 6.17:1 ratio. The AIN93-M group showed the highest food intake (Figure 3A). The daily consumption of chia flour was 2.14 g ± 0.14 (0.48 g of omega-3), and chia oil was 0.58 g ± 0.04 (0.46 g of omega-3) (Figure 3B). The highest final body weight was observed in the HFHF group. The addition of chia oil was able to reduce body weight at the AIN93-M level (Figure 3C). The Lee index was higher in the HFHF group, and chia flour and oil were able to reduce this parameter (Figure 3D).

### 3.3. Effect of Chia on Biochemical Parameters

Blood glucose and TG concentrations increased in the HFHF group and CO intake was able to reduce both parameters. The HFHF diet increased AST, ALT and uric acid levels, but CF and CO diets did not significantly reduce these levels (Table 2).

### 3.4. Gene Expression in the Liver

**Figure 4:**
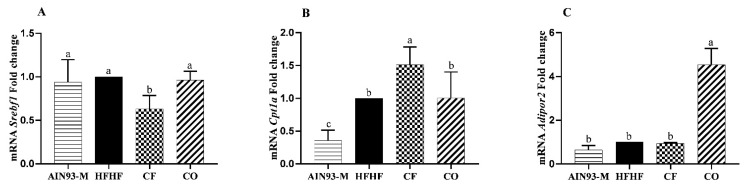
(**A**) *Srebf1:* sterol regulatory element-binding transcription factor 1; (**B**) *Cpt1a:* carnitine palmitoyltransferase 1a; (**C**) *Adipor2:* adiponectin receptor 2. The animals were fed diets: AIN93-M: standard rodent diet; HFHF: high-fat and high-fructose; chia flour (CF): HFHF with 4% of soybean oil replaced by chia flour lipid (14.7% of chia flour); and chia oil (CO): HFHF diet with 4% of soybean oil replaced by 4% of chia oil for 10 weeks. Values are represented by average and standard deviation (*n* = 10). The difference between the averages was analyzed by the one-way ANOVA test followed by the Newman–Keuls *post-hoc* test. Different letters indicate significant statistic difference (*p* < 0.05).

CF downregulated *Srebf1* gene expression (Figure 4A), as well as upregulated *Cpt1a* (Figure 4B). Otherwise, only CO upregulated *Adipor2* gene expression (Figure 4C).

### 3.5. Effect of Chia on Pro and Anti-Inflammatory Proteins in the Liver

**Figure 5:**
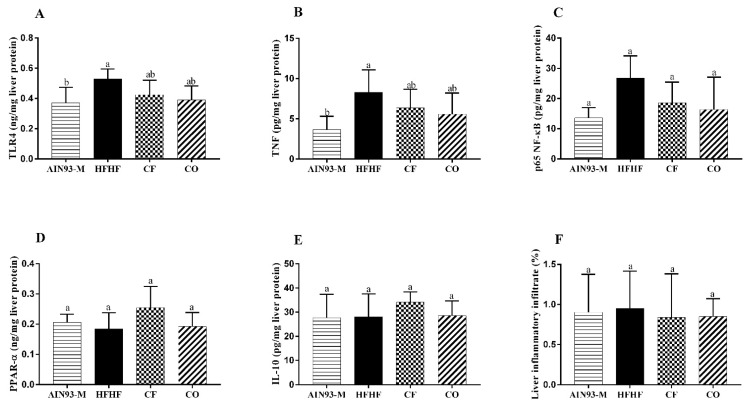
Effect of diets on inflammatory proteins and infiltrate in the liver at the end of 10 weeks. (**A**) Toll-like receptor 4 (TLR4); (**B**) tumor necrosis factor (TNF); (**C**) *p65*-nuclear factor kappa B transcriptional (NF-κB); (**D**) peroxisome proliferator-activated receptor alpha (PPAR-α); (**E**) interleukin 10 (IL-10); (**F**) liver inflammatory infiltrate. The animals were fed diets: AIN93-M: standard rodent diet; HFHF: high-fat and high-fructose; chia flour (CF): HFHF with 4% of soybean oil replaced by chia flour lipid (14.7% of chia flour); and chia oil (CO): HFHF diet with 4% of soybean oil replaced by 4% of chia oil for 10 weeks. Values are represented by average and standard deviation (*n* = 10). The difference between the averages was analyzed by the one-way ANOVA test followed by the Newman–Keuls *post-hoc* test. Different letters indicate significant statistic difference (*p* < 0.05).

The HFHF diet increased TLR4 and TNF levels compared to the AIN93-M diet. The consumption of CF and CO did not decrease these proteins, although chia treatments showed a similar pattern to the AIN93-M levels (Figure 5A,B). Regarding *p65*-NF-κB (Figure 5C), PPAR-α (Figure 5D), IL-10 (Figure 5E) levels and inflammatory infiltrate (Figure 5F), there were no significant differences among groups.

### 3.6. Effect of Chia on Oxidative Stress

**Figure 6:**
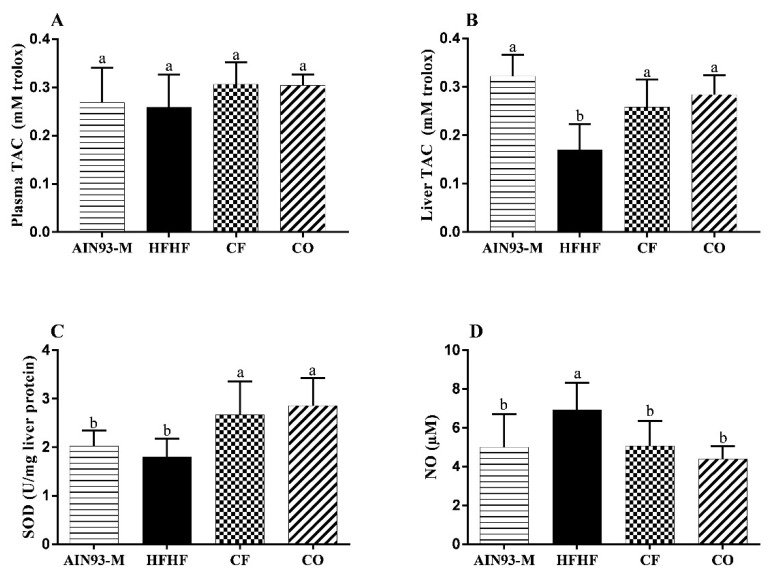
Effect of diets on oxidative stress. (**A**) plasma total antioxidant capacity (TAC); (**B**) liver total antioxidant capacity (TAC); (**C**) superoxide dismutase (SOD); and (**D**) nitric oxide (NO) in the liver. The animals were fed diets: AIN93-M: standard rodent diet; HFHF: high-fat and high-fructose; chia flour (CF): HFHF with 4% of soybean oil replaced by chia flour lipid (14.7% of chia flour); and chia oil (CO): HFHF diet with 4% of soybean oil replaced by 4% of chia oil for 10 weeks. Values are represented by average and standard deviation (*n* = 10). The difference between the averages was analyzed by the one-way ANOVA test followed by the Newman–Keuls *post-hoc* test. Different letters indicate significant statistic difference (*p* < 0.05).

No significant difference was observed in plasma TAC among experimental groups (Figure 6A). The analyses in the liver demonstrated that the chia consumption positively affected the TAC, restored the SOD activity, and reduced NO levels (Figure 6B–D).

### 3.7. Effect of Chia on Liver’s Biometric and Histomorphometry Parameters

**Figure 7:**
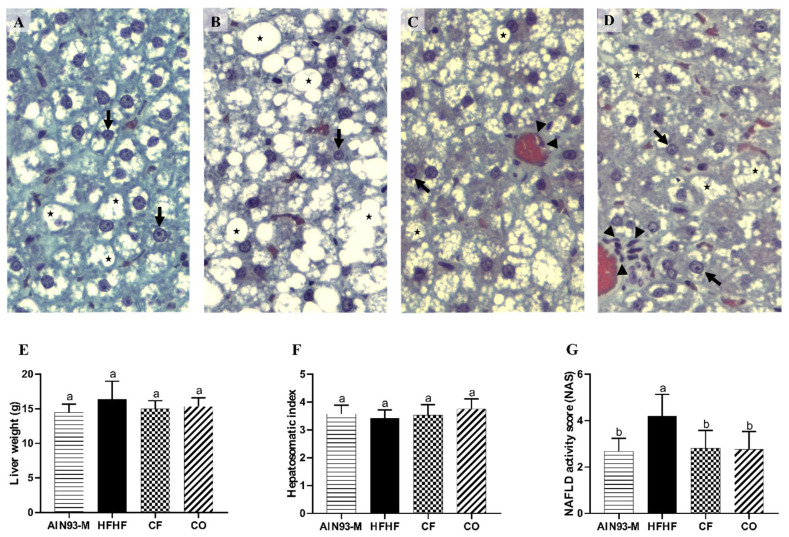
Representative histopathological photomicrographs of liver sections after 10 weeks od experiments in the groups: (**A**) AIN-93M (28.7% of lipid droplets in the liver); (**B**) HFHF: 4% of soybean oil, 31% lard, and 20% fructose) (animals showed 17.9% of lipid droplets in the liver); (**C**) chia flour: HFHF with 4% soybean oil replaced by chia flour (CF) lipid (14.7% of chia flour) (15.5% of lipid droplets in the liver); (**D**) chia oil (CO): HFHF with 4% of soybean oil replaced by 4% of chia oil (12.9% of lipid droplets in the liver); (**E**) liver weight; (**F**) hepatosomatic index; (**G**) NAFLD activity score (NAS). Gomori Trichrome Stain. (→) Hepatocyte nucleus; (>) lipid vesicles; (★) blood vessels. Bar = 50 μm. Values of liver weight (*n* = 10), hepatosomatic index (*n* = 8), and NAS (*n* = 8) are represented by average and standard deviation The difference between the averages was analyzed by the one-way ANOVA test followed by the Newman–Keuls *post-hoc* test. Different letters indicate significant statistical differences (*p* < 0.05).

Animals from the AIN93-M group retained 17.9% of lipid droplets in the liver, classified as liver steatosis grade 1 (Figure 7A), and the animals fed the HFHF diet showed liver steatosis grade 2 (28.7%—Figure 7B). CF and CO improved liver steatosis compared to the HFHF group (CF—15.5% and CO—12.9%) (Figure 7C,D). Among the groups, there was no significant difference in liver weight, hepatosomatic index (Figure 7E,F), and type I, III, and total collagen (data not shown). NAS was higher in the HFHF group (4.19), whereas AIN93-M, CF, and CO presented NAS of 2.68, 2.81, and 2.17, respectively (Figure 7G).

## 4. Discussion

The consumption of chia flour and chia oil improved liver steatosis and oxidative stress. In addition, flour and oil led to the downregulation and upregulation of genes involved in lipogenesis, and fatty acid oxidation, respectively. However, chia did not show significant effects on mitigating pro-inflammatory liver status in rats fed a high-fat and high-fructose (HFHF) diet. All these metabolic parameters evaluated in our study are recognized in NAFLD conditions.

Animals fed an HFHF diet demonstrated an unbalanced redox system evidenced by reduced TAC and increased NO levels in the liver. These findings could be explained by the lower antioxidants present in the HFHF diet, regarding its ingredients, impairing endogenous antioxidant levels. Moreover, long-term exposure to fructose and saturated fat provides a pro-oxidant environment by an increase in free fatty-acids oxidation, whereas lipid peroxidation leads to the production of lipotoxic species [30]. On the other hand, adding chia flour or chia oil into the HFHF diet improved the antioxidant system in the liver, observed by increased total antioxidant activity, increased SOD enzymatic activity levels, and reduced NO levels, which is a lipid peroxidation biomarker. Chia flour and oil contain antioxidant compounds, such as phenolic acids, flavonoids, peptides, vitamins (carotenoids and tocopherols), and minerals [10,11,31,32], which could assist in increasing antioxidant activity. Our results agree with the literature indicating chia has antioxidant properties in animal models. As described elsewhere, Wistar rats fed a high-fat diet (HFD) enriched with 41.3% of chia flour for five weeks increased the SOD (liver) and CAT (plasma) activities [33]. 

The high consumption of lipogenic substrates, such as fructose, promotes *de novo* lipogenesis regulated mainly by *Srebf1*, a gene involved in lipid biosynthesis which increases lipid retention in the hepatocytes [8]. In our study, the animals that consumed the HFHF diet showed 28.7% of fat accumulation in hepatocytes, which confirms that saturated fat and fructose are highly lipogenic. The animals AIN-93M showed 17.9% of lipid retention in the liver. Although AIN93-M is considered a standard diet for rodents, 77.4% of carbohydrates in its composition can lead to up-regulated *Srebf1* expression and trigger *de novo* lipogenesis [34]. It demonstrates the need to reassess and modify the carbohydrate content in the AIN93-M standard diet. Chia flour showed downregulation on *Srebf1*, upregulation on *Cpt-1a*, and reduction of 46% on lipid droplets in the liver compared to the HFHF group. *Cpt-1a* is involved in the fatty acids oxidation pathway, acting on the translocation of long-chain fatty acids from the cytosol to the mitochondrial matrix [5]. A study administered 36.2% of chia seed added into a sucrose rich diet (SRD) (24 weeks), and SRD plus 20% of chia seed (20 weeks) to Wistar rats and verified an increase of CPT-1 levels in the cardiac muscle and liver, respectively [35,36], which is a gene involved in lipogenesis process. An HFHF diet with 4% of chia oil (six weeks) and 13.3% of chia seed increased the receptor-γ coactivator 1-alpha (PGC-1α) protein levels in the skeletal muscle of Wistar rats, a biomarker involved in increasing beta-oxidation [37]. HFD plus 1.900 or 3.800 mg of CO/kg of body weight of mice for five weeks concomitantly reduced oxidative stress and liver steatosis [38]. While the dose of chia oil used in the mentioned study above is remarkably higher than our experiment, it can indicate that lower doses as used in our study are effective in reducing oxidative stress and liver fat content. Altogether, these findings indicate that chia act in different metabolic pathways assisting the reduction of metabolic liver disorders. The animals fed chia oil had upregulation on *Adipor2* gene expression in the liver, and reduction of 55% of liver lipid droplets compared to the HFHF group. In addition, chia oil was more efficient than chia flour in reducing blood glucose, plasma TG levels, and final body weight. The ADIPO-R2 is a receptor of adiponectin, an adipocytokine involved in lipid and glucose metabolism [39]. Although our present study did not quantify the levels of this protein, we hypothesize that the increased *Adipor2* expression may have played a key role in enhancing glucose uptake and inducing activation of pathways related to fatty acid oxidation. In our previous study, the addition of 4% of CO to Wistar rats fed the HFHF diet increased insulin sensitivity, recovering glucose metabolism by AMPK activation [16]. This enzyme can act on the lipid oxidation pathway, increasing mitochondrial beta-oxidation and consequent reduction of liver lipid droplets [40]. A study demonstrated that the administration of 0.15% of chia oil to male C57BL/6 mice for 45 days improved glucose metabolism [41,42]. Overall, our results demonstrate hepatoprotective properties of chia, attenuating the harmful effect caused by saturated fat and fructose and improving glucose uptake.

**Figure 8:**
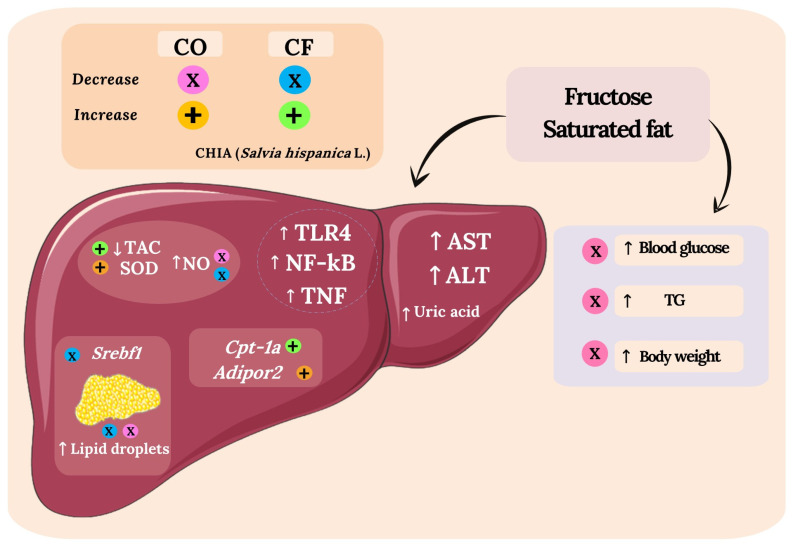
Metabolic disorders caused by the consumption of an HFHF diet, and the effects of chia flour (CF) and chia oil (CO) on the metabolic biomarkers evaluated in Wistar rats. The consumption of a high-fat and high-fructose (HFHF) diet caused metabolic disorders in the liver of the animals. The alterations were observed by the oxidant status, gene expression involved in lipogenesis, liver steatosis, and the activation of pro-inflammatory processes by increased TLR4, TNF, NF-κB, and protein levels in the liver. Furthermore, there was an increase in liver damage markers such as AST and ALT enzymes, and the uric acid concentrations, blood glucose, triglycerides (TG) levels, and body weight. The treatments with chia flour and chia oil increased SOD enzyme activity and reduced NO levels. The chia flour reduced lipogenesis and increased fatty acid oxidation observed by down-regulated *Srebf1*, and up-regulated *Cpt1a*, respectively, reducing liver steatosis. The chia oil up regulated *Adipor2*, a gene involved in lipid and glucose metabolism which decreased blood glucose, TG levels, liver steatosis, and body weight. No difference was observed in the PPAR-α, *p65*-NF-κB, and IL-10 proteins levels, demonstrating that the inflammation mitigation may occur later. Chia flour and chia oil added into the HFHF diet proved to be a relevant strategy to reduce metabolic disorders in the liver, parameters present in the NAFLD. *Adipor2:* adiponection receptor 2; ALT: alanine aminotransferase; AST: aspartate aminotransferase; CF: chia flour; CO: chia oil; *Cpt1a:* carnitine palmitoyltransferase 1a; HFHF: high-fat and high-fructose diet; NO: nitric oxide; *p65*-NF-κB: nuclear factor kappa-B transcriptional factor; SOD: superoxide dismutase; *Srebf1:* sterol regulatory element binding transcription factor 1; TAC: total antioxidant capacity; TG: triglycerides; TLR4: Toll-like receptor 4; TNF: tumor necrosis factor. ↑: increase; ↓: decrease; Chia effects: X-pink color: chia oil (CO) reduced NO, liver steatosis, blood glucose, TG and body weight; X-blue color: chia flour (CF) reduced NO, liver steatosis, and *Srebf1* expression; + in orange color: chia oil (CO) increased liver TAC, SOD activity, and *Adipor2* expression; + in green color: chia flour (CF) increased liver TAC, SOD activity, and *Cpt1a* expression.

The consumption of bioactive compounds such as omega-3 fatty acids and polyphenols can reduce the pro-inflammatory status and might lead to an anti-inflammatory effect [43]. One of these effects is the increase of PPAR-α levels. PPAR-α is a nuclear transcription factor that positively modulates the lipid metabolism acting as a regulator of free fatty acid oxidation, and its inactivation is also negatively involved with pro-inflammatory status, liver steatosis, mitochondrial dysfunction, and endoplasmic reticulum stress [44]. High levels of this protein can promote the inactivation and proteolytic degradation of *p65*-NF-κB, and consequently inhibit pro-inflammatory processes [45]. Our study demonstrated that the HFHF diet induced liver inflammation, observed by TLR4, TNF, and *p65*-NF-B proteins levels, without statistical differences among the groups regarding PPAR-α, IL-10 levels, and inflammatory infiltrates (Figure 5). Therefore, according to the results, we can claim that PPAR-α did not contribute to the reduction of liver steatosis. In addition, the consumption of chia flour and chia oil did not show any effect on IL-10 levels, an anti-inflammatory cytokine, as well as not reducing TLR4, *p65* NF-κB, and TNF protein levels. A study administered 0.15% of chia oil to rats fed HFD and there were no differences regarding plasma IL-10 [41]. The addition of 23.26% of chia flour into HFD (51% of fat) did not reduce NF-κB expression and TNF levels in female rat livers [46]. However, HFD plus 41.68% of chia flour offered to Wistar rats increased PPAR-α levels and reduced NF-κB and TNF levels in the liver [33]. It possibly could be due to a greater chia flour content. We might not have observed chia’s positive effect on liver inflammation due to the high fructose and saturated fat content in the HFHF diet, which led to a high pro-inflammatory state that may have blocked chia’s effect in this signaling pathway. Chia did not reduce the liver enzymes AST and ALT or uric acid, markers of liver damage caused mainly by the high consumption of fructose. Type I, III, and total collagen indicate liver fibrosis, and in this study, we did not observe a significant difference in these parameters. Probably, the duration of the experiment period was insufficient to induce fibrosis in the animal’s liver by the consumption of fructose. Concerning the NAFLD activity score, the highest NAS was observed in the HFHF group (Figure 7). Overall, according to our results, we can confirm that the HFHF diet induced metabolic disorders on the animal’s liver such as increased lipogenesis, liver steatosis, oxidative stress, and inflammation in the liver, features present in NAFLD. Nevertheless, chia flour and oil contributed to ameliorating these metabolic disorders in the liver. The beneficial effects of chia on liver health were observed through different mechanisms, and it is not directly linked to the resolution of liver inflammation (Figure 8), which indicates that the improvement in inflammation may occur later. We believe that the physical-chemical differences between chia flour and chia oil can be responsible for the different outcomes observed in this study. The chia oil (rich in ALA, lipophilic compounds, tocopherols, and phenols) led to up-regulated *Adipor2* expression, which improved glucose metabolism by reducing blood glucose, TG levels, and body weight. It also reduced oxidative stress and liver steatosis. In turn, chia flour may decrease ALA bioavailability and other lipophilic compounds since lipid enzymes may be more exposed in the chia oil rather than in chia flour. It can impact its digestion, absorption, transport, metabolism, and bioavailability. Therefore, the major components present in chia flour;dietary fibers, phenolic compounds, and micronutrients can be responsible for the downregulation of *Srebf1* combined with augmented *Cpt1a* expression, improvement of fatty acid oxidation and lipogenesis, as well as the redox system, and reduced liver steatosis.

## 5. Conclusions

The inclusion of chia flour or oil into an unbalanced diet rich in saturated fat and fructose promotes beneficial effects on liver health by reducing metabolic disorders through the modulation of different mechanisms. The chia flour and oil restored the antioxidant system and increased fatty acid oxidation. Furthermore, chia flour modulated lipogenesis, and chia oil improved blood glucose, TG levels, and body weight. These results reflected on reducing the degree of liver steatosis. Chia did not demonstrate a direct link to reduce liver inflammation, therefore it did not contribute to mitigating pro-inflammatory liver status, which requires further studies. In general, chia consumption is an interesting strategy for liver metabolic disorders.

## Figures and Tables

**Table 1:** Nutritional composition of experimental diets.

Ingredients (g/kg of Diet)	AIN93-M	HFHF	Chia Flour	Chia Oil
Albumin *	136.4	136.4	101.8	136.4
Dextrinized starch	155	45	45.4	45
Corn starch	463.5	135	116.8	135
Sucrose	100	28.6	29.3	28.6
Fructose	-	200	200	200
Soybean oil	40	40	-	-
Chia seed	-	-	147.3	-
Chia oil	-	-	-	40
Lard	-	310	310	310
Microcristalline cellulose	55.8	55.8	-	55.8
Mineral mix	35	35	35	35
Vitamin mix	10	10	10	10
L-cystine	1.8	1.8	1.8	1.8
Choline bitartrate	2.5	2.5	2.5	2.5
	Nutritional composition			
Macronutrients				
Carbohydrates (%)	77.4	30.1	31	30.1
Protein	12.9	9.1	9.2	9.1
Lipids	9.7	59.8	60.4	59.8
Energetic density (kcal/g)	3.71	5.26	5.21	5.26
Fatty acids (g/kg) **				
Linoleic (C18:2n−6)	20.2	58.8	46.5	46.5
α-Linolenic (C18:3n−3)	3.3	10.2	31.8	31.8
n−6/n−3 ratio	6.12:1	5.77:1	1.46:1	1.46:1

Experimental diets [16]: AIN93-M: standard rodent diet; and high-fat and high-fructose (HFHF); chia flour (CF): HFHF with 4% of soybean oil replaced by chia flour lipid (14.7% of chia flour); and chia oil (CO): HFHF diet with 4% of soybean oil replaced by 4% of chia oil for ten weeks. * Amount was calculated based on protein content equal to 88% to provide 12 g protein/100 g of diet. ** Fatty acids expressed in g/kg diet and determined by gas chromatography (Figure 2B).

**Table 2:** Effects of chia on biochemical parameters.

Experimental Diets				
Parameters	AIN-93M	HFHF	CF	CO
Glucose (mg/dL)	82.1 ± 12.1 ^b^	100.9 ± 7.8 ^a^	89.17 ± 2 ± 11.9 ^ab^	82.7 ± 11.5 ^b^
TG (mg/dL)	117.5 ± 17.1 ^b^	147.8 ± 21.6 ^a^	130.4 ± 7.8 ^ab^	115.8 ± 7.4 ^b^
AST (U/L)	98.5 ± 23.7 ^b^	150.7 ± 30.3 ^a^	172.0 ± 40.0 ^a^	172.9 ± 12.1 ^a^
ALT (U/L)	29.0 ± 11.1 ^b^	51.3 ± 16.9 ^a^	39.7 ± 6.3 ^ab^	52.1 ± 17.7 ^a^
Uric acid (mg/dL)	1.3 ± 0.7 ^b^	3.4 ± 2.4 ^a^	2.4 ± 1.2 ^ab^	1.7 ± 1.2 ^ab^

Animals fed AIN93-M: standard rodent diet; HFHF: high-fat and high-fructose; chia flour (CF): HFHF with 4% of soybean oil replaced by chia flour lipid (14.7% of chia flour); and chia oil (CO): HFHF diet with 4% of soybean oil replaced by 4% of chia oil for 10 weeks. Values are represented by average and standard deviation (*n* = 10). The difference between the averages was analyzed by the one-way ANOVA test followed by the Newman–Keuls *post-hoc* test. Different letters indicate significant statistic difference (*p* < 0.05). ALT: alanine aminotransferase; AST: aspartate aminotransferase; TG: triglycerides.

## Data Availability

Not applicable.
